# Comparison of Free Zinc Levels Determined by Fluorescent Probes in THP1 Cells Using Microplate Reader and Flow Cytometer

**DOI:** 10.1007/s12011-020-02355-w

**Published:** 2020-08-31

**Authors:** Wiebke Alker, Hajo Haase

**Affiliations:** 1 Food Chemistry and Toxicology, Technische Universität Berlin, Straße des 17. Juni 135, 10623 Berlin, Germany; 2 TraceAge - DFG Research Unit on Interactions of essential trace elements in healthy and diseased elderly, Potsdam-Berlin-Jena, Germany

**Keywords:** Free zinc, Flow cytometer, Microplate reader, Zinpyr-1, Fluozin-3 AM, Fluorescent probes

## Abstract

Free zinc is involved in signal transduction within mammalian cells, acting as a second messenger. Gold standard for its analysis is currently the use of metal-responsive fluorescent probes. The present study elucidates the impact of instrumentation used for measuring the resulting fluorescence. The free zinc concentration of THP-1 cells loaded with the fluorescent probes Zinpyr-1 (ZP1) or Fluozin-3 AM (FZ3) was determined using a microplate reader (MPR) and a flow cytometer (FC). Depending on the instrumentation, either low nanomolar (MPR) or picomolar (FC) concentrations of free zinc were observed. The concentrations measured from identical samples by MPR were about 40 (ZP1) or 165 (FZ3) times higher compared with FC. These results demonstrate that the choice of instrumentation has a fundamental impact on the determination of intracellular free zinc concentrations by low molecular weight fluorescent probes.

## Introduction

Zinc ions play a crucial role in numerous signaling pathways. Changes in free zinc concentrations are involved in intercellular communication and act as a second messenger for transmitting information within cells [1, 2]. In this context, the determination of the intracellular free zinc concentration is of great significance.

Fluorescent probes are useful tools for detection and quantification of metal ions. Their functional principle is based on the binding of the analyte to a metal-specific binding site, inducing an alteration of the optical properties of an attached fluorophore. For detailed information on fluorescent metal ion sensors, the reader is referred to review articles on this subject (e.g., [3, 4]). For zinc, the two cell-permeable and zinc-selective fluorescent probes Zinpyr-1 (ZP1) and Fluozin-3 AM ester (FZ3) are commonly used for determining the free zinc concentration in cells. Here, the term “free zinc” describes the zinc pool that is not tightly bound to proteins, but relatively weakly bound to low molecular weight ligands [5, 6]. In order to quantify the free zinc concentration, the following equation by Grynkiewicz et al. can be applied for non-ratiometric probes such as FZ3 and ZP1 [7]:

$$ \left[{Zn}^{2+}\right]={K}_{\mathrm{D}}\cdotp \frac{F-{F}_{\mathrm{min}}}{F_{\mathrm{max}}-F} $$

Herein, *K*_D_ indicates the dissociation constant of the zinc:probe complex, *F* represents the fluorescence of the probe induced by free zinc present in the cell, *F*_min_ the autofluorescence of the probe in the absence of zinc, and *F*_max_ the maximum fluorescence of the zinc-saturated probe.

The fluorescence intensity is typically measured by either one of two different types of instruments, microplate readers (MPR) or flow cytometers (FC). This study examines whether the resulting free zinc concentrations are comparable when different techniques are used.

## Materials and Methods

### Materials

Bovine serum albumin (BSA) (Sigma-Aldrich, Germany); Dulbecco’s Modified Eagles Medium (DMEM) (PAN-Biotech, Germany); fetal calf serum (FCS) (CCPro, Germany); Fluozin-3 AM ester (FZ3) (Thermo Fisher Scientific, USA) N,N,N′,N′-tetrakis(2-pyridinylmethyl)-1,2-ethanediamine (TPEN) (Sigma-Aldrich, Germany); Zinpyr-1 (ZP1) (Santa Cruz Biotechnology, USA); ZnSO_4_ • 7 H_2_O (Sigma-Aldrich, Germany). All other chemicals were purchased from standard sources.

### Cell Culture

THP-1 cells (obtained from Leibniz Institute DSMZ-German Collection of Microorganisms and Cell Cultures GmbH, Germany) were cultured in DMEM, containing FCS (10%), penicillin (100 U/mL), and streptomycin (100 μg/mL) at 37 °C, 5% CO_2_, and humidified atmosphere. The medium was changed every two to three days.

### Fluorescence Staining and Incubation of the Cells

THP-1 cells were seeded into 96-well plates (2 × 10^5^ cells/per well in 200 μL assay buffer; 120 mM NaCl, 5.4 mM KCl, 5 mM glucose, 1 mM CaCl_2_, 1 mM MgCl_2_, 1 mM NaH_2_PO_4_, 10 mM HEPES, pH 7.35), centrifuged (5 min at 166 rcf) and the supernatant removed. Subsequently, cells were incubated with incubation buffer (assay buffer with 0.3% BSA), either 200 μL containing 0.5 μM ZP1 or 50 μL containing 1.0 μM FZ3, for 30 min at 37 °C, 5% CO_2_, and humidified atmosphere. Cells were then washed once with assay buffer to remove extracellular probe before addition of 200 μL assay buffer alone (F), or supplemented either with the chelator TPEN (1–100 μM) or the ionophore sodium pyrithione (NaPyr, 25 μM) and ZnSO_4_ (1–500 μM) to induce *F*_min_ or *F*_max_, respectively. Cells were incubated for additional 30 min at 37 °C, 5% CO_2_, and humidified atmosphere before measuring fluorescence intensity.

### Measurement of Fluorescence Intensity

Fluorescence intensity was first measured by MPR (ZP1: Infinite M200, Tecan, Switzerland, FZ3: SPARK, Tecan, Switzerland) (ZP1: λ_ex_ 492 nm, λ_em_ = 527 nm; FZ3: λ_ex_ 490 nm, λ_em_ = 515 nm). Subsequently, 96-well plates were directly transferred into the FC (CytoFLEX, Beckman Coulter, Germany) and single-cell fluorescence measured at λ_ex_ 488 nm and a band-pass filter at 525/40 nm.

## Results and Discussion

**Fig. 1:**
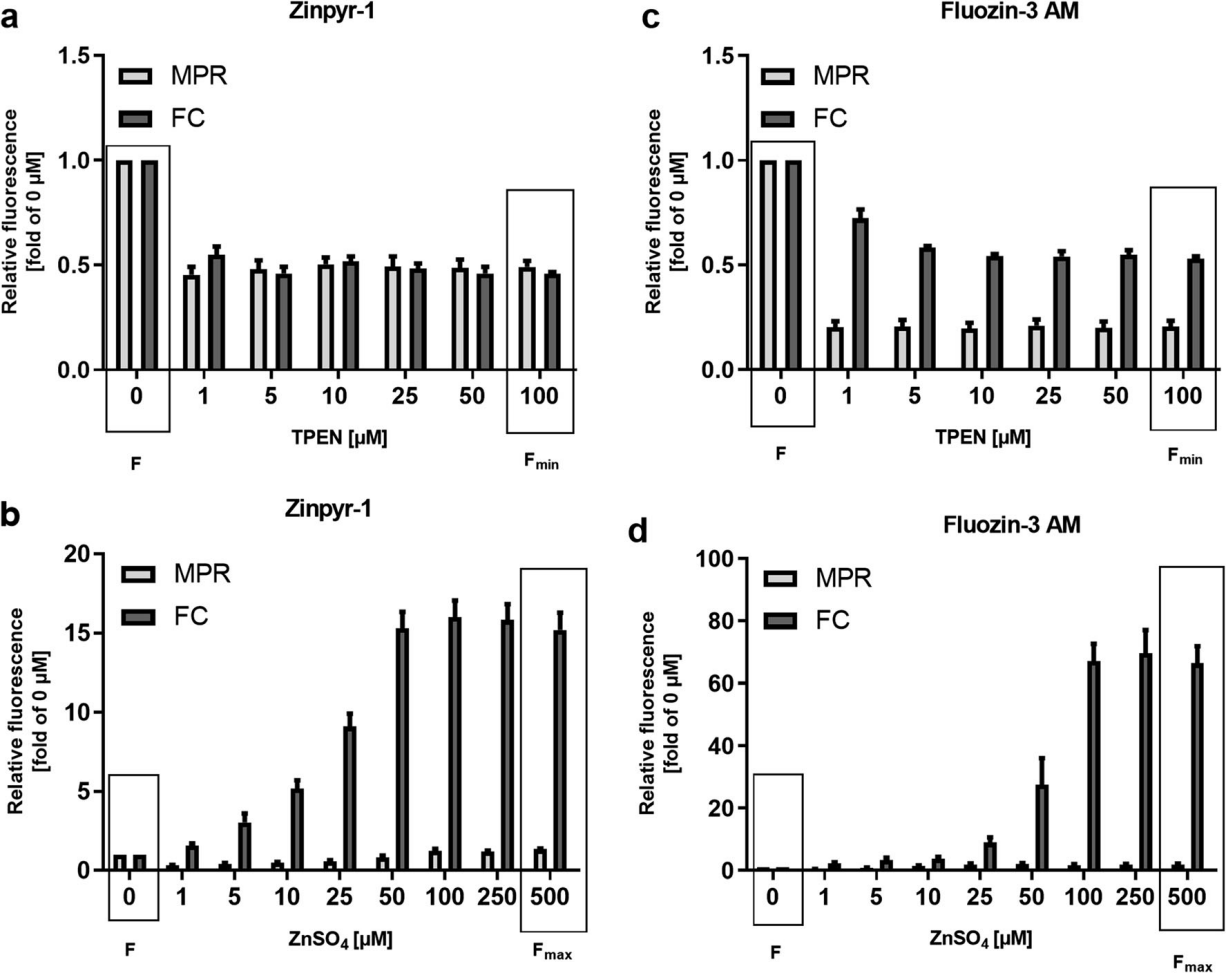
Relative fluorescence intensity of fluorescent probe–loaded THP-1 cells measured by MPR and FC. THP-1 cells were incubated with **a**, **b** ZP1 (0.5 μM) or **c**, **d** FZ3 (1.0 μM) in incubation buffer before addition of either 200 μL assay buffer alone (0 μM) or containing **a**, **c** 1–100 μM TPEN or **b**, **d** 25 μM NaPyr and 1–500 μM ZnSO_4_. Cells were incubated for 30 min before read-out of fluorescence intensity by MPR and FC. Data are shown relative to *F* (fluorescence intensity of 0 μM ZnSO_4_) and represent means + SD of *n* = 3 independent experiments

The apparent free zinc concentration of THP-1 cells varies considerably depending on the instrument used for measuring zinc-dependent fluorescence. For ZP1, fluorescence intensity values measured by MPR (1.05 ± 0.30 nM) yielded about 40 times higher values for free zinc compared with FC (0.027 ± 0.006 nM) even though identical samples were investigated. Applying the equation by Grynkiewicz et al., the calculated result is influenced by the relative ratios of *F* to *F*_min_ and *F*_max_ to *F* [7]. To induce *F*_min_, the zinc chelator TPEN was used. For both instruments, concentrations from 1 to 100 μM resulted in a comparable ratio of *F*_min_ to *F* of about 0.5 (Fig. 1a). In contrast, the ratio of *F*_max_ to *F* differs between MPR and FC (Fig. 1b). A higher ratio, as in the case of the FC, results in lower calculated free zinc concentrations.

Comparable observations were made with another zinc-selective probe, FZ3. Free zinc determined by MPR (10.38 ± 5.37 nM) was 165 times higher than by FC (0.063 ± 0.012 nM). As for ZP1, the ratio of *F*_max_ to *F* is substantially higher when measured by FC (Fig. 1d). In addition, the ratio of *F*_min_ to *F* also differs slightly between approximately 0.2 for MPR and 0.5 for FC (Fig. 1c), contributing to the difference of the calculated free zinc concentrations between the two instruments as a lower ratio of *F*_min_ to *F* results in higher calculated values for free zinc.

In addition to the variance between different instruments, the calculated free zinc concentrations also differ between ZP1 and FZ3, when these are measured on the same device. Even though the probes have different affinities for zinc, with FZ3 being the lower affinity probe (K_D_ 8.9 nM) compared with ZP1 (K_D_ 0.7 nM) [8, 9], comparable values would have to be expected. However, this only applies if both probes localize to the same intracellular compartments. It has repeatedly been shown that this is not the case for ZP1 and FZ3 [10, 11], so the present data do not allow an evaluation of the effect that the choice of different fluorescent probes may have on the resulting free zinc concentration. Notably, in human serum, where compartmentalization is not an issue, FZ3 and ZP1 yield comparable free zinc values, indicating that both probes do give according results (data not shown).

The effect has been observed with two different zinc-selective fluorescent probes, indicating that it is not a particularity of ZP1 and FZ3, but more likely a general observation that is relevant for other probes, as well, not even being limited to zinc-selective probes. Accordingly, different ratios of fluorescence intensity values between MPR and FC for fluorescent probe–loaded cells, after inducing fluorescence intensity by identical treatment, have also been described by Pasquier et al. analyzing human breast cancer cells with the fluorescent probe Calcein acetoxymethyl ester. The ratio relative to untreated control cells is reported to be higher by FC than by MPR, and the difference between the two measurement approaches amplified with increasing fluorescence intensity [12].

To our knowledge, parallel measurements of free zinc by MPR and FC in the same samples have not been previously reported. Still, a higher calculated free zinc concentration resulting from measurements by MPR compared with FC seems to be a recurring pattern in the literature. The results of different studies that are comparable with regard to their experimental setup typically show nanomolar free zinc concentrations by MPR, while FC yields sub-nanomolar concentrations. In HL-60 cells incubated with FZ3, Dubben et al. determined a free zinc concentration of 1.75 ± 0.61 nM using an MPR, whereas Wessels et al. reported approximately 0.04 nM using a FC [13, 14]. In monocytes and lymphocytes from peripheral venous blood incubated with FZ3, the calculated free zinc concentrations were approximately 2.5 nM and 4.4 nM determined by MPR, compared with 0.17 ± 0.06 nM and 0.35 ± 0.07 nM by FC, respectively [15, 16].

**Fig. 2:**
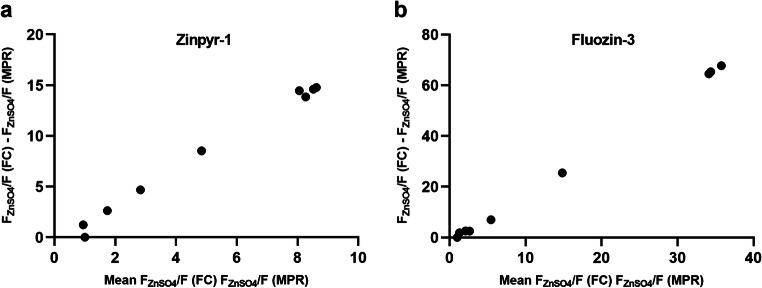
Difference between zinc-induced increase in fluorescence intensity by FC and MPR plotted against the mean. THP-1 cells were incubated with **a** ZP1 (0.5 μM) or **b** FZ3 (1.0 μM) in incubation buffer before addition of either 200 μL assay buffer alone (*F*) or containing 25 μM NaPyr and 1–500 μM ZnSO_4_ (F_ZnSO4_). Cells were incubated for 30 min before read-out of fluorescence intensity by MPR and FC. Data represent means of *n* = 3 independent experiments

In the present study, identical single-cell suspensions were analyzed by two different instruments; therefore, the cause of the observed differences must lie in the instrumentation. When comparing the results of two methods, Bland and Altman suggest plotting the differences against the mean as this allows conclusions about the comparability of the methods [17]. As *F* represents the fluorescence intensity induced by free intracellular zinc and F_ZnSO4_ the fluorescence intensity induced by incubation with increasing zinc concentrations, the value for *F* is constant while F_ZnSO4_, and thereby the ratio of F_ZnSO4_ to *F*, increases when ZnSO_4_ is added. In Fig. 2, a proportional increase of the ratios of F_ZnSO4_ to *F* determined by MPR and FC would lead to a constant relation between the difference of the ratios determined by both methods (depicted on the y-axis) to their means (shown on the x-axis). This is not the case for either fluorescent probe. There is a smaller increase of F_ZnSO4_ by MPR than by FC, indicating a decreasing sensitivity of the MPR for higher fluorescence intensity, compared with FC. Such an observation has also been made by Pasquier et al. in a comparable context [12] and has to be considered when trying to explain the observed discrepancies.

**Fig. 3:**
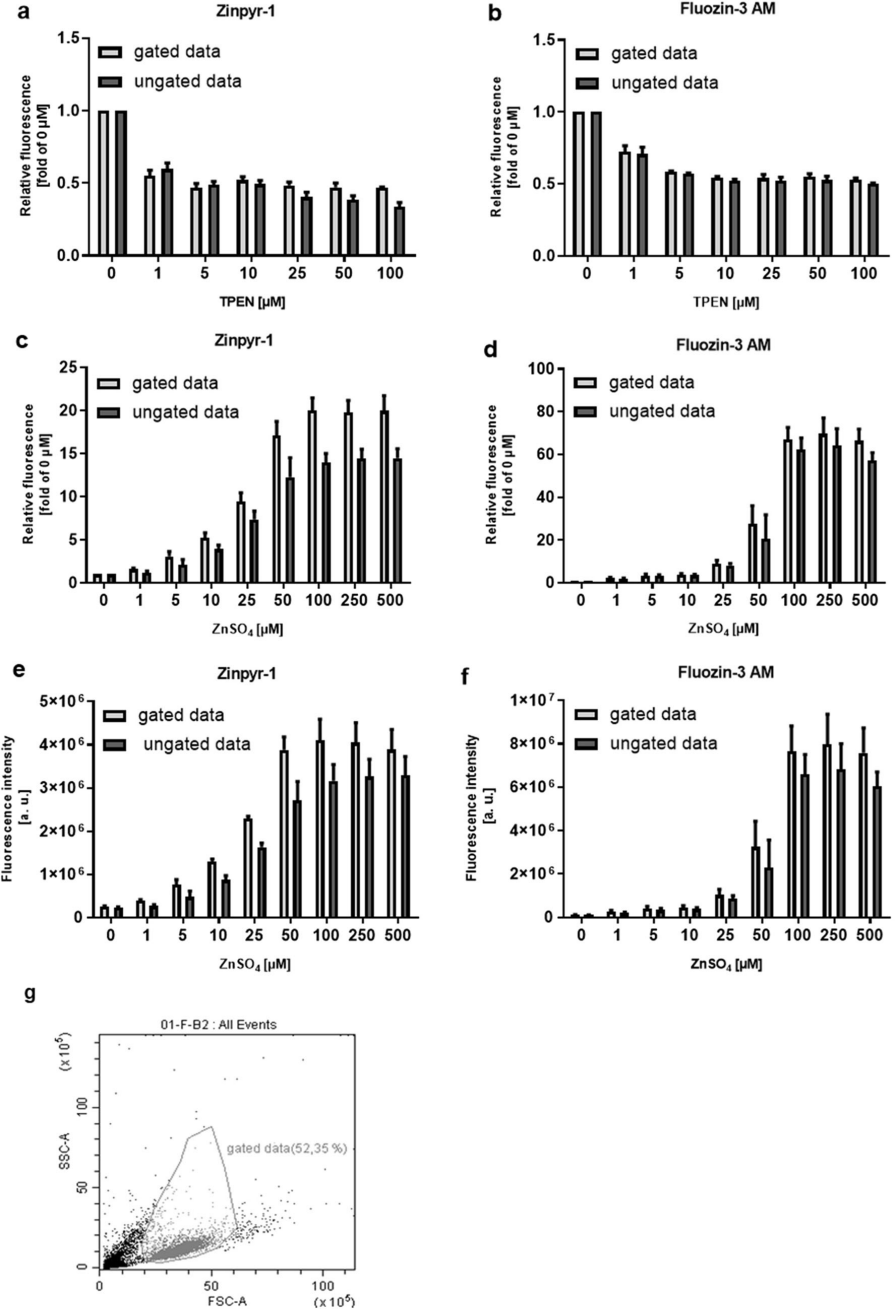
Comparison of different data processing of fluorescence intensity values of fluorescent probe–loaded THP-1 cells measured by FC. THP-1 cells were incubated with ZP1 (0.5 μM) (**a**, **c**, **e**, **g**) or FZ3 (1.0 μM) (**b**, **d**, **f**) in incubation buffer before addition of either 200 μL assay buffer alone (0 μM) (**g**) or containing 1–100 μM TPEN (**a**, **b**) or 25 μM NaPyr and 1–500 μM ZnSO_4_ (**c**, **d**, **e**, **f**). Cells were incubated for 30 min before read-out of fluorescence intensity by FC. Data are shown relative to *F* (fluorescence intensity of 0 μM TPEN or ZnSO_4_) (**a**, **b**, **c**, **d**) or as fluorescence intensity in arbitrary units (**e**, **f**) and represent means + SD of *n* = 3 independent experiments or as dot plot, showing forward scatter (Fsc) and sideward scatter (Ssc) (**g**). Ungated data include all measured events (**g**, black and gray events), and gated data include selected cells only (**g**, gray events)

An MPR light passes through the single-cell suspension and fluorescence intensity is recorded as the sum of zinc-bound ZP1 molecules within a well. Neither during data recording nor data processing it is possible to gain any further information, such as size or intactness of the respective cells that are source of the fluorescence signal. On the other hand, in a FC, the cells pass a laser one by one. In addition to fluorescence intensity, information about size and granularity is measured and can be assigned to each recorded event. As a result, it is possible to select cells of interest for subsequent data processing, which is a common procedure for flow cytometry and often referred to as gating, shown as an example in Fig. 3 g. Subsequent analysis is then based on fluorescence intensity values from these gated cells only, instead of all measured events. This had been performed with the FC data shown in Fig. 1 to exclude cell debris.

Using fluorescence signals from all events measured by the FC instead of gated data results in higher calculated free zinc concentrations for ZP1 (0.037 ± 0.006 nM instead of 0.027 ± 0.006 nM) and FZ3 (0.077 ± 0.012 nM instead of 0.027 ± 0.006 nM). For both probes, the ratio of *F*_min_ to *F* is comparable between gated and ungated data (Fig. 3a, d), whereas the ratio of *F*_max_ to *F* is smaller without gating (Fig. 3b, e). This ratio decreases either when the value for *F* gets bigger or the value for *F*_max_ gets smaller. Here, the absolute values for both *F* and *F*_max_ are smaller for ungated datasets (Fig. 3c, f). Inclusion of cell debris, which would otherwise be eliminated by the gating process, results in a lower median of ungated fluorescence intensity values compared with the gated dataset. Proportionally, the decrease of *F*_max_ is bigger than the decrease of *F*, so the smaller ratio of *F*_max_ to *F* in ungated data is caused by a smaller *F*_max_. Excluding cell debris in FC might therefore contribute to the observed differences between both instruments. However, this effect due to data processing is relatively small compared with the overall differences in calculated free zinc concentrations observed between MPR and FC, and cannot be seen as the sole reason for this discrepancy. As FC measures individual cellular fluorescence, while MPR records bulk fluorescence within a well, the latter would also be prone for measuring cell fragments too small to be recorded by FC, at all, and even extracellular fluorescence. Cells were washed to remove extracellular dye, but still probe could be released through leakage during the experiment, which might be a source of considerable perturbation for MPR measurements. Finally, differences in the excitation and detection of the fluorescence signal, based on the different technological setups, may also contribute to the differences in fluorescence intensity ratios and resulting free zinc concentrations when comparing MPR and FC.

## Conclusion

Both MPR and FC can be used to detect the fluorescence intensity of cells incubated with zinc ion-sensitive fluorescent probes. For some experiments, the choice between MPR and FC is of lesser importance, e.g., when only trends or relative changes are investigated. Here, aspects such as instrument availability, sample throughput, or coefficient of variation can be taken as decisive factors. However, when these values are used for quantification of the intracellular free zinc concentration, the choice of instrumentation critically impacts the obtained values. This factor also needs to be considered when comparing literature results obtained using different measurement approaches.

The most important question, which instrument gives a better representation of the actual free zinc concentration, remains unsolved. The present study cannot provide a definitive answer, as the true intracellular zinc concentrations are not known. Because MPR includes data from debris and damaged cells, as well as it might pick up extracellular fluorescence from leakage, it seems that the lower, sub-nanomolar values obtained by FC might be more reliable. These are also a better match for values obtained by other methods not based on fluorescent probes [18, 19], as well as the sub-nanomolar affinities of many cellular zinc-binding proteins [20].

## Data Availability

Data will be made available upon request.
